# GWAS and RNA-seq reveal novel loci and genes of low-nitrogen tolerance in cucumber (*Cucumis sativus* L.)

**DOI:** 10.3389/fpls.2025.1602360

**Published:** 2025-06-06

**Authors:** Huaxiang Wu, Huiming Yan, Bowen Li, Yike Han, Nan Liu, Mengyu Fan, Yahan Liu, Mingjie Lyu, Shengli Du, Aimin Wei

**Affiliations:** ^1^ College of Life Science, Nankai University, Tianjin, China; ^2^ Cucumber Research Institute, Tianjin Academy of Agricultural Sciences, Tianjin, China; ^3^ State Key Laboratory of Vegetable Biobreeding, Tianjin Academy of Agricultural Sciences, Tianjin, China; ^4^ Institute of Germplasm Resources and Biotechnology, Tianjin Academy of Agricultural Sciences, Tianjin, China

**Keywords:** nitrogen use efficiency, GWAS, RNA-Seq, VIGS, CsGATA9

## Abstract

Cucumber (*Cucumis sativus* L.), a globally significant horticultural crop, requires substantial nitrogen inputs due to its high nutrient demand. However, the prevalent issues of low nitrogen use efficiency (NUE) in cultivars and excessive fertilizer application have led to increased production costs and environmental burdens. To identify quantitative trait nucleotides (QTNs) and genes associated with low-nitrogen tolerance, we conducted a genome-wide association study (GWAS) on a basis of three low-nitrogen tolerance traits and 594,066 single nucleotide polymorphisms (SNPs) of a natural population of 107 cucumber accessions. The transcriptome of low-nitrogen tolerant genotype (F005) and low-nitrogen sensitive genotype (F027) were sequenced between low and normal nitrogen treatments. Through GWAS, we identified 29 QTNs harboring 196 candidate genes, while RNA sequencing (RNA-seq) revealed 3,765 differentially expressed genes (DEGs). 24 were identified by both methods. Among these 24 genes, 20 genes showed significant phenotype differences among different haplotypes. These 20 genes were defined as more valuable candidate genes for low-nitrogen tolerance. Furthermore, functional validation of the candidate gene *CsaV3_7G035390* (encoding a GATA9 transcription factor) was performed using virus-induced gene silencing (VIGS), which demonstrated that silencingn this gene significantly enhanced soil plant analysis development (SPAD) and leaf of nitrogen accumulation in cucumber, indicating its negative regulatory role in low-nitrogen tolerance. Collectively, this study provides novel genetic resources for improving NUE in cucumber breeding programs.

## 1 Introduction

Nitrogen is a vital element for life, playing a critical role in plant growth, yield, and stress tolerance. For instance, when plants experience nitrogen deficiency, they exhibit several characteristic symptoms, including stunted growth, pale yellowing leaves, reduced branching, and decreased yield (de Bang et al., 2021). Furthermore, nitrogen metabolism is essential for plant stress resistance, as it regulates ion balance, reduces reactive oxygen species (ROS) production, promotes chlorophyll synthesis, and maintains normal photosynthesis (Soualiou et al., 2023). However, in agricultural practice, excessive nitrogen fertilization exacerbates environmental burdens and promotes the cultivation of nitrogen-sensitive cultivars. This issue is especially prominent in facility vegetable cultivation, where intensive cropping systems and continuous cropping have led to elevated nitrate levels. As one of the most widely cultivated crops in facilities, cucumbers are particularly susceptible due to their shallow root systems and strong preference for both water and nitrogen (Hua et al., 2022). Therefore, understanding the molecular mechanisms underlying low-nitrogen tolerance in cucumbers, along with identifying QTNs and key genes associated with low-nitrogen tolerance, represents a promising strategy for achieving sustainable development in the cucumber industry.

Nitrogen regime classification is critical for stress phenotyping and genetic analysis in plants, as nitrogen demands vary across growth stages and genotypes, requiring concentrations that distinguish nitrogen-responsive phenotypes without causing irreversible physiological damage. In cereal crops like wheat, low nitrogen (LN) is defined as 70–144 kg N ha^-^¹ and normal nitrogen (NN) as 170–206 kg N ha^-^¹, thresholds that reduce biomass by 20% while maintaining plant viability (Cormier et al., 2013). For hydroponic systems, nitrogen concentrations are often optimized based on modified Hoagland nutrient solutions: for example, Rapeseed (*Brassica napus* L.) uses LN (a quarter of Hoagland solution, 3.75 mM NO_3_^-^) and NN (full Hoagland solution, 15 mM NO_3_^-^), where LN reduces aboveground biomass by 24-80% (Ahmad et al., 2022); watermelon seedlings employ LN (0.75 mM NO_3_^-^) and NN (7.5 mM NO_3_^-^) to characterize root growth and nitrogen acquisition traits under controlled conditions (Zhang et al., 2025). In cucumber, previous transcriptomic studies on genotypes with contrasting low-nitrogen tolerance validated LN (3 mM NO_3_^-^) and NN (14 mM NO_3_^-^) as effective concentrations to differentiate nitrogen-responsive gene expression without compromising plant survival (Xin et al., 2021). Building on these standards, our study defines LN as 3.5 mM nitrate and NN as 14 mM nitrate in hydroponic cultures, aligning with both the physiological thresholds established in cucumber and recent GWAS research that validated these concentrations for phenotypic and genomic analysis of nitrogen stress tolerance.

Regarding nitrogen status evaluation, handheld chlorophyll meters, such as SPAD meters, have proven to be valuable tools for the rapid, non-destructive assessment of chlorophyll content and nitrogen levels in various crops. This method is frequently employed to diagnose the need for nitrogen fertilization, ultimately improving agricultural efficiency and minimizing nitrogen losses and deficiencies. For example, Li et al. (2022a) assessed nitrogen-deficiency tolerance (NDT) in 230 rice accessions by measuring SPAD in flag leaves under two nitrogen levels. Their study revealed significant genetic differences between indica and japonica subspecies, with greater SPAD variation observed under nitrogen-deficient conditions. Similarly, plant height (PH) is commonly used as a key phenotypic indicator to evaluate plant growth and nitrogen response. Wang et al. (2022) employed both PH and SPAD as shoot traits to conduct a GWAS analysis on maize under low-nitrogen stress. In parallel, Lv et al. (2021) identified four phenotypes, including PH, as the main low-nitrogen-induced growth response traits in 225 rice accessions (PH, tiller number, chlorophyll content, and leaf length). In addition, shoot dry weight (SDW) can be used as indicators for evaluating low-nitrogen tolerance in oat varieties (Wang et al., 2023a) sorghum (Liu et al., 2020a), and soybeans (Guo et al., 2024).

Recent genomic studies have significantly expanded our understanding of the genetic mechanisms that regulate nitrogen utilization and tolerance in cucumbers. For instance, key regulatory factors such as *CsbZIP55* and *CsbZIP65* have been identified through whole-genome analyses, quantitative real-time PCR (qRT-PCR) analysis, and transcriptional activation experiments (Hua et al., 2023). Moreover, RNA interference (RNAi) targeting *CsIVP* has been shown to enhance cucumber plants’ resilience to both nitrogen deficiency and high-temperature stress (Yan et al., 2022). Amino acid transporters also play a vital role in organic nitrogen transport and plant growth. For example, Yao et al. (2023) revealed that extracellular amino acid accumulation in the roots of *CsAAP2* mutants could disrupt the pH balance of the apoplast, thereby affecting auxin synthesis and its distribution within roots. Furthermore, overexpression of *CsGS1* significantly enhanced LN tolerance and improved photosynthetic parameters, chlorophyll b content, biomass, PH, root length, nitrogen accumulation, and glutamine synthetase (GS) activity under LN (Xin et al., 2021). GWAS and transcriptomic analysis has proven effective in identifying QTNs and key genes associated with low-nitrogen tolerance in cucumbers. In a related study, Li et al. (2023a) conducted a GWAS analysis on 88 cucumber accessions under low-nitrogen treatment and identified 9 significant loci and 5 genes associated with low-nitrogen tolerance. Additionally, RNA-seq technology has proven invaluable in identifying nitrogen-responsive genes across various plant species, including rice (Zhang et al., 2024; Wang et al., 2023b; Subudhi et al., 2020), wheat (Kaur et al., 2022; Wang et al., 2021a; Sultana et al., 2020; Zhang et al., 2021), and Arabidopsis (Qiao et al., 2022).

The integration of GWAS and transcriptomics has emerged as a powerful strategy for deciphering the genetic architecture of complex traits. For instance, in rice, the combined application of GWAS and RNA-seq uncovered *OsHTAS*-mediated ROS-hormone crosstalk mechanisms underlying heat tolerance (Li et al., 2023b). Similarly, multi-omics approaches in cotton identified *GhAMT2* as a central regulator of Verticillium wilt resistance through GWAS-transcriptomics integration (Wang et al., 2025). Notably, such integrative GWAS-RNA-seq frameworks have also demonstrated considerable potential in mining heat tolerance candidate genes in cotton, as evidenced by recent advancements (Luqman et al., 2025). These achievements demonstrate that GWAS efficiently locates trait-associated loci, while transcriptomic dynamics reveal spatiotemporal specificity in gene expression regulation, providing multidimensional evidence chains for functional gene discovery. However, cucumber low-nitrogen tolerance research remains limited to single-omics approaches, lacking systematic integration of genetic variation with dynamic gene expression networks.

In this study, we performed a GWAS based on phenotypic data (PH, SPAD, and SDW) and 594,066 SNPs generated from resequencing 107 cucumber accessions. Subsequently, transcriptomic profiling of two contrasting genotypes (low-nitrogen tolerant F005 and sensitive F027) was conducted to dissect their transcriptional dynamics under nitrogen deprivation. Through multi-omics integration, 196 candidate genes were initially identified within 50-kb flanking regions of QTNs, while transcriptomic profiling revealed 3,765 low-nitrogen-responsive genes. Subsequent intersection analysis and haplotype analysis mapped 20 high-confidence candidates. By integrating VIGS technology, we verified that downregulation of *CsGATA9* expression significantly promotes SPAD and leaf of nitrogen accumulation under LN. The findings from this study providing a theoretical foundation for identifying low-nitrogen response genes and improving low-nitrogen tolerance in cucumbers.

## 2 Materials and methods

### 2.1 Plant materials and phenotype evaluation

A total of 107 cucumber accessions (88 from our core collection (Li et al., 2023a) and 19 newly introduced) obtained from the Tianjin Academy of Agricultural Sciences (China) were subjected to hydroponic cultivation for GWAS: seeds were presoaked in 55°C water, germinated at 28°C in darkness, acclimatized in a phytotron (25°C, 7 days), and transplanted into rectangular boxes (59 × 38 × 14.5 cm) containing half-strength Hoagland solution; after 9 days, uniform seedlings were exposed to LN (2.5 mM NO_3_¯ + 1 mM NH_4_^+^;) and NN (13 mM NO_3_¯ + 1 mM NH_4_^+^;) treatments for 14 days, with triplicate measurements of PH, SPAD (SPAD-502Plus, KONICA MINOLTA), and SDW (oven-dried at 105°C/30 min followed by 65°C/72 h). For RNA-seq analysis, two extreme genotypes (F005: low-nitrogen tolerant; F027: low-nitrogen sensitive) identified were cultivated in vermiculite-filled pots under controlled humidity (60–80%): germinated seeds received purified water irrigation for 4 days, followed by half-strength Hoagland solution for 9 days prior to LN/NN treatments (10 days), with all experiments conducted in triplicate to ensure reproducibility. All accessions were cultivated at the experimental station of the Tianjin Academy of Agricultural Sciences, which is located in Wuqing, Tianjin, China (39°25’N, 117°02’E). The detailed concentrations of elements in the various nutrient solutions are provided in the **Supplementary Material** (**Supplementary Table 1**). To maintain nutrient availability, the nutrient solutions were refreshed every five days.

### 2.2 RNA sequencing and data analysis

Total RNA was extracted from LN and NN treated leaf tissues using TRIzol^®^ reagent (Invitrogen, USA). Polyadenylated mRNA was enriched through oligo(dT) magnetic bead selection and converted into strand-specific RNA-seq libraries via fragmentation, first-strand cDNA synthesis, and PCR amplification. Libraries were sequenced on an Illumina NovaSeq 6000 platform (LC-Bio, China) with 150 bp paired-end configuration. Raw reads were quality-filtered using cutadapt (v1.9) with stringent parameters: adapter trimming (-a/-A), quality trimming (Phred score < 20), and length filtering (-m 100). High-quality reads were aligned to the Cucumis sativus reference genome (v3; Li et al., 2019a) via HISAT2 (v2.0.4) (Kim et al., 2015) with default splice-junction detection settings. Transcript abundance was quantified using StringTie (v1.3.4d) (Pertea et al., 2015) in reference-guided mode (-G annotation.gtf), with expression levels normalized as fragments per kilobase of transcript per million mapped reads (FPKM) through Ballgown (v2.40.0) (Frazee et al., 2015). Differential gene expression analysis was performed using edgeR (v4.6.1) (Robinson et al., 2010) with generalized linear models. Genes exhibiting |log_2_(fold change)| ≥ 1 and pvalue < 0.05 were defined as DEGs. Gene ontology (GO) analysis was performed using the online platform OmicShare (https://www.omicshare.com/tools), as outlined by (Mu et al., 2024). The top 20 items (*P* value < 0.023) were considered to be the most significantly enriched biological processes.

### 2.3 Genotyping and data filtering

The 19 newly introduced cucumber accessions were subjected to whole-genome resequencing (Illumina NovaSeq 6000 platform) by Novogene Co. (Beijing, China). Genomic DNA was extracted from leaf tissues using the TIANGEN^®^ Plant DNA Secure Kit (DP320, China). Sequencing libraries were prepared with the Illumina TruSeq Nano DNA Library Prep Kit (San Diego, CA) following manufacturer protocols. Raw sequencing data from these accessions were merged with existing genomic data of 88 cultivars (Li et al., 2023a). The raw data were subjected to a filtration process to discard reads that harbored over 50% low-quality bases (quality value < 5), in excess of 10% unidentified bases (N), as well as any adaptor contamination. Processed reads were mapped to the Cucumis sativus v3 reference genome (Li et al., 2019a) using BWA-MEM (v0.7.8) (Li and Durbin, 2009) with parameters -t 4 -k 32 -M. SAMtools (v1.3) was employed for BAM file sorting (sort) and PCR duplicate removal (rmdup), achieving a mean mapping rate of 82.3% and average sequencing depth of 18.45× (range: 10.1–25.3×). The SNPs were called using GATK software (McKenna et al., 2010) and filtered by VCFtools (v0.1.16) (Danecek et al., 2011) with parameters max-missing 0.9, maf 0.05, minDP 2, maxDP 1000, minQ 30, minGQ 0, min-alleles 2, and max-alleles 2. Collectively, 594,066 high-quality SNPs were amassed for subsequent analysis.

### 2.4 Population characteristics and linkage disequilibrium analysis

The population structure was evaluated by ADMIXTURE (v1.23) (Alexander et al., 2009) investigate the population structure with the number of assumed genetic clusters K ranged from 1 to 10, and with subgroups assigned according to delta K value. FastTree (v2.1) (Price et al., 2010) was used to construct a phylogenetic tree using the maximum likelihood method. Principal component analysis (PCA) was carried out using Plink (v1.9) software (Purcell et al., 2007). LD decay analysis to identify candidate regions was performed using PopLDdecay (v3.42) (Zhang et al., 2019). The average *R* ^2^ values of pairwise SNP markers were calculated for all SNPs in the genome, and the candidate region was identified where average *R* ^2^ decreased to half of the maximum value.

### 2.5 GWAS analysis

GWAS was carried out utilizing the recently developed 3VmrMLM model (Li et al., 2022b) on a genetic panel consisting of 107 cucumber accessions and 594,066 SNPs. In the case of PH, the association values were computed based on the nitrogen response value RN_PH, which was obtained as RN_PH = (LN_PH_ − NN_PH_)/NN_PH_. Here, LN_PH_ denotes the PH under LN, and NN_PH_ represents the PH under NN. Analogously, for the SPAD, the association values were calculated in accordance with the nitrogen response value RN_SPAD, which was derived as RN_SPAD = (LN_SPAD_ − NN_SPAD_)/NN_SPAD_, and RN_SDW= (LN_SDW_ − NN_SDW_)/NN_SDW_. This methodology effectively accentuates the genetic responses of cucumber accessions to low-nitrogen stress, facilitating the identification of crucial loci that contribute to NUE and related traits. The R package “IIIVmrMLM” was utilized to identify main-effect QTNs associated with RN_PH RN_SPAD, and RN_SDW. The parameters for main-effect QTNs detection were configured as follows: method = Single_env, SearchRadius = 20, and svpal = 0.01. The population structure (Q) matrix was calculated using admixture with k = 4, while the kinship (K) matrix was obtained from the “IIIVmrMLM” package. Marker-trait associations were established by applying a threshold of LOD score ≥ 3.

### 2.6 Identification of candidate genes

Candidate genes related to low-nitrogen tolerance were identified through GWAS. Putative candidate genes were located within 50 kb upstream and downstream of the main-effect QTNs by means of BEDTools (v2.31.0) software (Quinlan and Hall, 2010). To identified key genes response to low-nitrogen, the candidate genes were selected following three standards: (1) localization within QTN regions; (2) differential expression in LN vs. NN treatments of F005/F027; (3) significant haplotype effects (*P* value < 0.05) on traits of RN_PH/RN_SPAD/RN_SDW. Based on functional annotations, expression differences, and haplotype analysis, key candidate genes were selected for further verification. The haplotype analysis centered on SNPs from promoter regions (defined as the 1-kb upstream sequence from the transcription start site) and intragenic regions. Haplotype analysis was conducted using the R package geneHapR (Zhang et al., 2023).

### 2.7 VIGS analysis

To verify the function of the identified candidate gene *CsGATA9*, a VIGS system was employed. A 400-bp coding sequence of *CsGATA9* was amplified using specific primers: forward primer 5′-GTGCGATGATTTAGCGGAACTC-3′ and reverse primer 5′-CTTCTCCGCCTGACAATGCA-3′. The fragment was cloned into the *Sna*BI restriction site of the pTRV2 vector via homologous recombination and transformed into Agrobacterium tumefaciens strain GV3101. The transformed Agrobacterium cultures were grown overnight in Luria-Bertani liquid medium supplemented with appropriate antibiotics at 28°C, and then resuspended in an induction buffer containing 10 mM MES and 200 µM acetosyringone. When the primary roots of germinating cucumber seeds (F005 genotype) reached 1 cm in length, the seeds were vacuum infiltrated at 0.09 MPa for 8 minutes with a mixture of pTRSV1 and pTRSV2 vectors at a 1:1 ratio. The seeds were placed on half-strength Murashige and Skoog solid medium containing 10 mM MES and 200 μM acetosyringone until the presence of Agrobacterium was visible around the seeds. Seedlings were then transferred into half-strength Hoagland nutrient solution and grown for approximately three weeks until TRSV2: *CsPDS* whitening was observed. Leaf tissues from plant of TRSV: 00 and TRSV: *CsGATA9* groups were collected for qRT-PCR analysis. Total RNA was extracted using an RNA extraction kit (Hlinggene, Shanghai, China) and reverse-transcribed into cDNA using a Reverse Transcription Kit (Lablead, Fuzhou, China). Gene expression levels were measured using SYBR Green Master (Yeasen, Shanghai, China) and calculated using the 2−ΔΔCt method (Livak and Schmittgen, 2001), with *CsACTIN* as the internal control (forward primer: 5′-ATCGTGGTGATGTTGTGCCT-3′; reverse primer: 5′-AGCAACACTGGTGGAGTTGG-3′). Plants with a silencing efficiency greater than 70% were considered gene-silenced lines and subjected to subsequent LN treatment. After two weeks under LN, PH, SPAD, and leaf of nitrogen content (%) were measured. PH was measured with a ruler, SPAD was quantified using a chlorophyll meter (SPAD-502Plus, KONICA MINOLTA, Inc., Japan), and nitrogen content (%) was determined using a high-temperature combustion method with an elemental analyzer (EA3100, Euro Vector, Italy) for both TRSV:00 and TRSV: *CsGATA9* groups. These data were used to analyze the phenotypic effects and validate the role of *CsGATA9* in low-nitrogen tolerance.

### 2.8 Statistical analysis

Statistical analyses were conducted using the Student’s *t*-tests, as implemented in GraphPad Prism software. Significance levels were indicated by asterisks, with *, ** and *** representing differences at *P* < 0.05, *P* < 0.01 and *P* < 0.001, respectively.

## 3 Results

### 3.1 Phenotypic analysis of PH, SPAD and SDW in response to low-nitrogen tolerance

**Figure 1:**
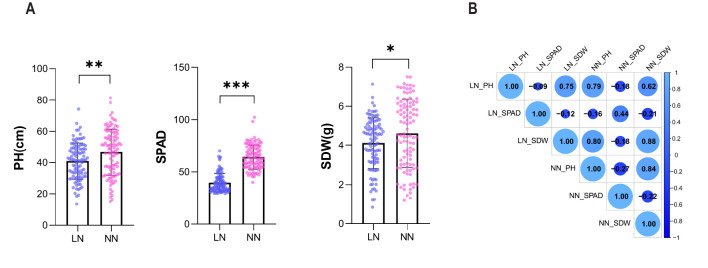
Phenotypic analysis of PH, SPAD and SDW in response to LN. **(A)** PH, SPAD and SDW for 107 cucumber accessions under LN andNN. *** denote significance at P < 0.001, ** at P < 0.01, and * at P < 0.05. **(B)** Correlation analysis of PH, SPAD and SDW. The size of the circles represents the correlation coefficient, and the gradient color indicates the direction and strength of the correlation (positive or negative).

In this study, a phenotypic analysis was conducted on 107 cucumber accessions to evaluate PH, SPAD and SDW under LN and NN. As shown in **Figure 1**, all three traits (PH, SPAD, and SDW) exhibited significant reductions under LN compared to NN conditions. The mean of PH, SPAD and SDW for the LN were 40.81 cm, 39.49 cm, and 4.01 g, whereas under the NN, these values were 45.91 cm, 64.72 cm, and 4.47g. The standard deviations for PH, SPAD and SDW under LN were 11.16, 8.65 and 1.29, compared to 14.60, 11.95 and 1.72 under NN (**Table 1**). Additionally, a significant negative correlation between PH and SPAD was observed under both LN and NN, with correlation coefficients of −0.09 under LN and −0.27 under NN. Notably, a significant negative correlation of SDW with SPAD (−0.12 under LN, −0.22 under NN) while positive with PH (0.75 under LN, 0.84 under NN) (**Figure 1B**).

**Table 1:** Descriptive statistics of PH, SPAD and SDW under LN and NN.

Trait	Mean	Maximum	Minimum	SD
LN_PH	40.81	74.28	13.50	11.16
LN_SPAD	39.49	70.27	29.07	8.65
LN_SDW	4.01	7.14	0.84	1.29
NN_PH	45.91	81.42	15.20	14.60
NN_SPAD	64.72	102.25	39.43	11.95
NN_SDW	4.47	7.52	1.19	1.72
RN_PH	-0.07	0.76	-0.50	0.22
RN_SPAD	-0.38	0.06	-0.60	0.13
RN_SDW	-0.06	0.69	-0.52	0.21

LN_PH, PH under LN; LN_SPAD, SPAD under LN; LN_SDW, SDW under LN, NN_PH, PH underNN; NN_SPAD, SPAD under NN; NN_SDW, SDW under NN; RN_PH, the nitrogen response value of RN_PH, which was derived as RN_PH = (LN_PH_ – NN_PH_)/NN_PH_; RN_SPAD, the nitrogen response value of RN_SPAD, which was derived as RN_SPAD = (LN_SPAD_ – NN_SPAD_)/NN_SPAD_; RN_SPAD, the nitrogen response value of RN_SPAD, which was derived as RN_SDW = (LN_SDW_ – NN_SDW_)/NN_SDW_; Max, Maximum; Min, Minimum; SD, std. devt; PH, measured in cm; SDW measured in g.

### 3.2 Population structure and LD decay analysis

**Figure 2:**
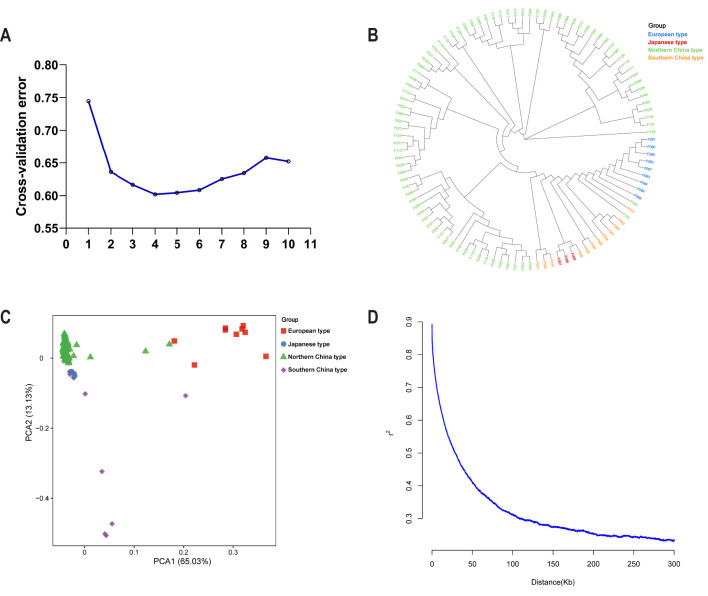
Population structure and LD decay analysis of the association panel consisting of 107 accessions. **(A)** Cross-validation error values for different numbers of clusters (K=1-10) based on genotype data. **(B)** Phylogenetic trees constructed based on maximum likelihood method. **(C)** PCA of the 107 accessions, with different colors representing different groups: Eurasian (red squares), Japanese (green circles), North Chinese (green triangles), and South Chinese (purple diamonds). **(D)** LD decay analysis of the 107 accessions.

The population structure of the 107 accessions was analyzed using several complementary methods. Initially, the optimal number of clusters (K) was determined by calculating the cross-validation error values for K ranging from 1 to 10 (**Figure 2A**). The cross-validation error reached a minimum at K=4, indicating that four clusters best represent the population structure of the association panel. Phylogenetic analysis further elucidated the relationships among the 107 accessions (**Figure 2B**). The phylogenetic tree revealed distinct clades corresponding to the geographical origins of the accessions, with clear separation between the European, Japanese, Northern China, and Southern China types, which showed the diversity of cucumber accessions. PCA provided additional insights into the population structure (**Figure 2C**). The first two principal components (PC1 and PC2) explained 65.03% and 13.13% of the total genetic variation, respectively. The PCA plot demonstrated a clear separation of the accessions, consistent with their geographical origins. The European type (red squares) formed a distinct cluster, while the Japanese type (green circles), the Northern China type (green triangles), and the Southern China type (purple diamonds) were also well-separated. LD decay analysis was conducted to evaluate the extent of LD within the association panel (**Figure 2D**). The LD decay curve illustrated that r² decreased rapidly with increasing physical distance. By applying the criterion of LD decay distance, defined as the physical distance at which the average LD coefficient declines to half of its maximum value, we observed that the average LD coefficient reached this threshold at approximately 50 kb. This rapid LD decay indicates a high level of recombination and genetic diversity present within the association panel. In summary, the population structure analysis revealed distinct genetic clusters corresponding to the geographical origins of the accessions, while the LD decay analysis indicated a significant level of genetic diversity within the panel. These findings provide a solid foundation for subsequent GWAS and other genetic analyses.

### 3.3 GWAS identified Main-effect QTNs and candidate genes

**Figure 3:**
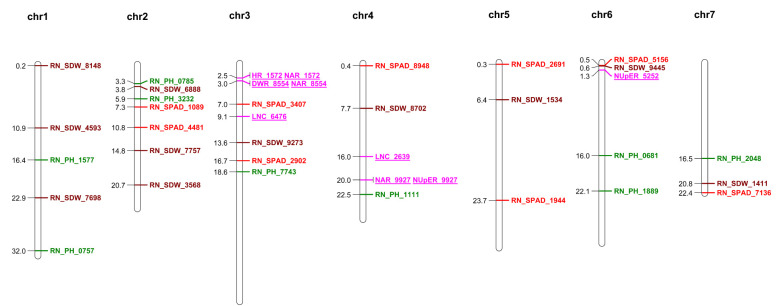
Chromosomal distribution of main-effect QTNs associated with RN_PH, RN_SPAD and RN_SDW. The numbers on the left of a chromosome indicate the physical locations of the corresponding main-effect QTNs, measured in Mb. Main-effect QTNs associated with RN_PH are marked in green, RN_SPAD are marked in red, and RN_SDW are marked in brown. QTNs previously reported in published literature are marked in purple and underlined (Li et al, 2023a).

In this study, we conducted a GWAS to identify main-effect QTNs and their associated genes related to RN_PH, RN_SPAD and RN_SDW. Utilizing the 3VmrMLM model, we analyzed 594,066 SNPs across 107 cucumber accessions, revealing key genetic loci that influence these important traits. A total of 29 main-effect QTNs were identified; of these, RN_PH was associated with 9 QTNs, accounting for 69.72% of the phenotypic variation, RN_SPAD was associated with 9 QTNs that explained 69.13% of the phenotypic variation, and RN_SDW was associated with 11 QTNs that explained 55.23% of the phenotypic variation (**Table 2**). Furthermore, the QTN located at chr7_22371357, associated with RN_SPAD, exhibited the largest r² and LOD values. Although the QTNs identified in this study are not identical to those reported in previous studies, several QTNs were found in close proximity to previously reported loci (RN_SPAD_3407 near LNC_6476 on chr3, RN_PH_1111 near NAR_9927/NUpER_9927 on chr4, and RN_SPAD_5156 and RN_SDW_9445 near NupER_5252 on chr6) (**Figure 3**). This close proximity suggests that these loci may belong to the same LD block or represent overlapping regulatory regions. Such results support the reliability of our findings, as they partially validate previous studies while also identifying novel QTNs that were not detected in prior research. A total of 196 genes were identified within the QTN regions, comprising 65 associated with RN_PH, 62 with RN_SPAD and 69 with RN_SDW (**Supplementary Table 2**). Notably, the gene *CsaV3_2G013230*, which is homologous to *At5G43700*, is considered a key player in nitrogen metabolism (Gaudinier et al., 2018). The research demonstrated through yeast one-hybrid experiments that IAA4 regulates essential processes in nitrogen metabolism, including nitrogen transport, assimilation, and signaling. Furthermore, we also identified a growth regulator, *CsaV3_6G000720*, which is similar to a growth regulator factor (GRF). GRFs are an important transcription factor family in plants, with GRF4 shown to regulate multiple nitrogen metabolism genes and interact with DELLA proteins, enhancing cereal yield and exacerbating dwarfism in rice (Li et al., 2018). These findings suggest that genes from the GRF family may promote adaptive responses to LN by regulating nitrogen metabolism-related gene expression. These consistent research results with previous studies indicate the reliability of GWAS.

**Table 2:** Main-effect QTNs associated with the traits of RN_PH, RN_SPAD and RN_SDW detected in 107 cucumber accessions.

QTNs name	Trait	Chromosome	Position	LOD	variance	r^2^(%)	P-value
RN_SDW_8148	RN_SDW	chr1	168148	4.292	0.0014	3.3208	5.11E-05
RN_SDW_5932	RN_SDW	chr1	10945932	6.2983	0.0018	4.2901	7.22E-08
RN_PH_5768	RN_PH	chr1	16415768	11.9907	0.0050	10.4745	1.08E-13
RN_SDW_6980	RN_SDW	chr1	22876980	10.0697	0.0021	4.7877	8.53E-11
RN_PH_7565	RN_PH	chr1	32007565	6.7266	0.0032	6.6681	1.88E-07
RN_PH_0785	RN_PH	chr2	3290785	6.5938	0.0032	6.5906	2.55E-07
RN_SDW_6888	RN_SDW	chr2	3766888	7.3876	0.0026	6.106	4.10E-08
RN_PH_3232	RN_PH	chr2	5883232	8.892	0.0044	9.2013	1.56E-10
RN_SPAD_1089	RN_SPAD	chr2	7271089	9.112	0.0013	7.4846	9.31E-11
RN_SPAD_4805	RN_SPAD	chr2	10844805	3.2135	0.0005	2.8818	1.20E-04
RN_SDW_5702	RN_SDW	chr2	14775702	4.4637	0.0015	3.4588	3.44E-05
RN_SDW_5683	RN_SDW	chr2	20735683	5.9514	0.0013	3.1006	1.65E-07
RN_SPAD_3407	RN_SPAD	chr3	7043407	7.1409	0.0012	6.7425	7.23E-08
RN_SDW_2728	RN_SDW	chr3	13592728	6.5902	0.0022	5.196	2.57E-07
RN_SPAD_9016	RN_SPAD	chr3	16729016	5.9264	0.0007	4.2146	1.75E-07
RN_PH_7433	RN_PH	chr3	18577433	5.0991	0.0024	5.0559	7.96E-06
RN_SPAD_8948	RN_SPAD	chr4	368948	6.1673	0.0006	3.375	9.86E-08
RN_SDW_8702	RN_SDW	chr4	7698702	11.3788	0.0038	8.7074	4.53E-13
RN_PH_1107	RN_PH	chr4	22511107	8.1782	0.0042	8.7084	6.64E-09
RN_SPAD_2691	RN_SPAD	chr5	282691	8.5069	0.0013	7.2181	3.11E-09
RN_SDW_1534	RN_SDW	chr5	6401534	4.8351	0.0016	3.5991	1.46E-05
RN_SPAD_9438	RN_SPAD	chr5	23719438	10.0582	0.0013	7.5385	8.75E-11
RN_SPAD_5156	RN_SPAD	chr6	465156	13.2414	0.0026	14.4581	5.78E-15
RN_SDW_9445	RN_SDW	chr6	629445	7.4952	0.0027	6.2755	3.20E-08
RN_PH_6807	RN_PH	chr6	16006807	6.9138	0.0033	6.8065	1.22E-07
RN_PH_8892	RN_PH	chr6	22118892	8.817	0.0046	9.5245	1.53E-09
RN_PH_0481	RN_PH	chr7	16520481	15.7609	0.0032	6.6938	1.60E-17
RN_SDW_4109	RN_SDW	chr7	20814109	9.9489	0.0028	6.392	1.13E-10
RN_SPAD_1357	RN_SPAD	chr7	22371357	30.8431	0.0027	15.2159	1.44E-31

variance, the variance of each QTN; r^2^ (%), the proportion of total phenotypic variance explained by each QTN; P-value, calculated from LOD score using χ2 distribution.

### 3.4 Transcriptional analysis of low-nitrogen tolerant and low-nitrogen sensitive cucumber genotypes under low-nitrogen tolerance

**Figure 4:**
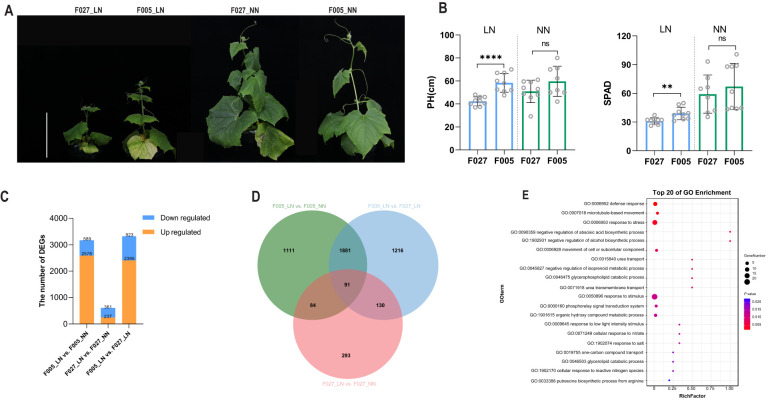
DGEs between two genotypes (F005, F027) under LN and NN. **(A)** Phenotypic presentation of F027 and F005 after nitrogen treatment. The scale bar represents 16 cm. **(B)** PH and SPAD for F027 and F005 after nitrogen treatment, with **** indicating significance at *P* < 0.0001, ** denote significance at *P* < 0.01, and “ns” indicating no significant difference. **(C)** Numbers of up- and downregulated DEGs under LN and NN conditions in F005 and F027. **(D)** Venn diagram analysis of line F005 and F027 under LN and NN. **(E)** GO analysis of 91 DEGs in **(D)**, the size of the circles represents the number of genes, while the color gradient indicates the magnitude of the *P* value.

Typically, F027 showed a remarkable growth inhibition, whereas F005 exhibited a slight growth inhibition under LN. Significantly lower PH and SPAD for F027 compared to F005 under LN whereas, no significant differences were observed under NN (**Figure 4A-B**). F005 demonstrated a higher capacity for nitrogen accumulation under LN in previous study, consistent with its observed higher PH and SPAD under these conditions. The strong tolerance exhibited by F005 may be attributed to more efficient nitrogen utilization mechanisms. To further explore transcriptional differences under LN, we analyzed the expression profiles of leaf tissues from two parental lines (F005 and F027) under LN and NN. After filtering out low-quality sequences and adapters, an average clean dataset of 6.43 gigabases per sample was obtained, with a Q30 mean of 97.70%. The average unique mapping rate across all samples was 83.2% (**Supplementary Table 3**). DEGs in leaves were identified by comparing two nitrogen levels (F005_LN vs. F005_NN and F027_LN vs. F027_NN) and two genotypes (F005_LN vs. F027_LN) (**Supplementary Tables 4**-**6**). The analysis revealed distinct gene expression profiles induced by LN in F005 and F027. Specifically, F005 exhibited a greater number of upregulated genes, whereas F027 showed a higher number of downregulated genes. This significantly impacted nitrogen metabolism-related pathways and genes. In contrast, when comparing LN to NN, 3,167 DEGs were regulated in F005 and 598 in F027. Moreover, 3,318 DEGs were identified exclusively in both genotypes under LN, surpassing other combination comparisons (**Figure 4C**). These results suggested that DEGs identified under LN, distinguishing low-nitrogen tolerant and low-nitrogen sensitive cucumber genotypes, merit further investigation. Venn diagram analysis (**Figure 4D**) revealed unique and overlapping gene sets between the two accessions under different nitrogen conditions. Notably, 91 common genes were identified at the intersection of the three groups, which are likely crucial in the response to LN, and *CsNRT2.5* was pinpointed in the differential comparison groups of all three groups and exhibited upregulated expression across all groups (**Supplementary Table 7**). GO enrichment analysis of these 91 genes indicated significant enrichment in biological processes and molecular functions associated with the LN response, including nitrogen metabolic regulation, and amino acid synthesis and transport (**Supplementary Table 8**, **Figure 4E**). These findings imply that the low-nitrogen tolerant genotype F005 employs enhanced transcriptional regulation to mitigate nitrogen limitation. These insights provide a new understanding of the tolerance mechanisms in cucumbers under LN stress and offer potential molecular targets for future research aimed at improving nitrogen efficiency in crops.

### 3.5 Screening key genes for low-nitrogen tolerance and the functional identification of *CsGATA9*

**Figure 5:**
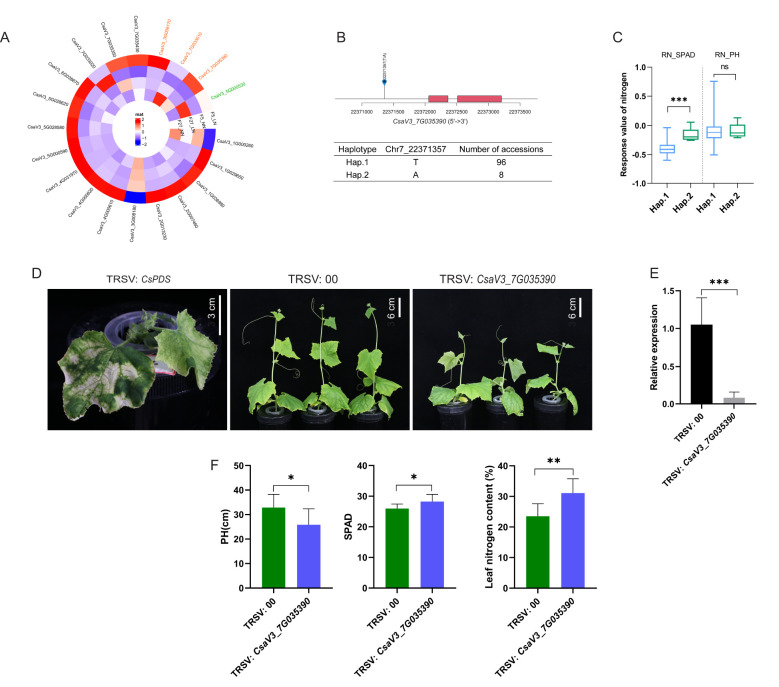
Candidate genes and functional validation of *CsGATA9* by VIGS. **(A)** Heatmap showing the expression levels (FPKM) of candidate genes. The genes ID marked black across F005_LN vs. F005_NN, marked green across F027_LN vs. F027_NN, the overlap genes of two group marked orange. **(B)** Haplotype analysis of *CasV3_7G035390* (*CsGATA9*), showing the expanded upstream 1 kb region and the distribution of different haplotypes (Hap. 1 and Hap. 2). **(C)** Boxplot showing the phenotypic performance of the two haplotypes of *CsGATA9* in RN_SPAD and RN_PH. (***P<0.001 and “ns” indicating no significant difference). **(D)** Phenotypic differences among TRSV: *Cs*PDS, TRSV: 00, and TRSV: *CsGATA9* cucumber plants under low-nitrogen treatment for two weeks. TRSV: *CsPDS* plants, used as a positive control, exhibited typical chlorotic leaves, indicating the effectiveness of the TRSV system. TRSV: *CsGATA9* plants showed reduced growth compared to TRSV:00 plants. **(E)** Relative expression levels of *CsGATA9* in leaves of TRSV:00 and TRSV: *CsGATA9* plants, measured by qPCR 14 days after viral inoculation. A significant reduction in *CsGATA9* transcript levels confirms successful gene silencing (***P < 0.001). **(F)** Quantitative comparison of PH, SPAD and leaf of nitrogen content(%) between TRSV:00 and TRSV:*CsGATA9* plant sunder LN. Plant height in TRSV:*CsGATA9* plants was significantly reduced, while SPAD values and nitrogen content of leaf(%) were significantly increased compared to TRSV:00 plants (*P<0.05 and **P<0.01). Each bar represents the mean ± SD of three independent experiments, with three biological 867 replicates per experiment (n=3). Statistical significance was determined using Student’s t-test.

The GWAS analysis identified 196 genes associated with RN_PH, RN_SPAD and RN_SDW. Additionally, RNA-seq analysis across two comparisons (F005_LN vs. F005_NN and F027_LN vs. F027_NN) revealed a total of 3765 DEGs. Notably, 24 genes overlapped between the DEGs and the GWAS-identified genes. According to the haplotype analysis of 24 genes, 20 genes showed significant phenotype differences among different haplotypes (**Table 3**). Among these genes, 16 genes were differential expressed in the comparisons of F005_LN vs. F005_NN, only 1 gene in F027_LN vs. F027_NN, 3 genes were differential expressed in the both comparisons. *CsaV3_7G035390* and *CsaV3_7G033010* were upregulated in the comparisons of F005_LN vs. F005_NN but downregulated in the comparisons of F027_LN vs. F027_NN. Conversely, *CsaV3_3G008170* was consistently upregulated across both two comparisons (**Figure 5A**). *CsaV3_7G035390* was mapped to chromosome 7 (Chr7) between genomic positions 22371588 and 22373765 bp, encoding a GATA9 transcription factor. Haplotype analysis of the GATA9 transcription factor revealed a SNP that resulted in two haplotypes among the 107 accessions: Hap.1 (T) and Hap.2 (A). Significant phenotypic differences in RN_SPAD were observed between two haplotypes (**Figure 5B-C**). To investigate the potential role of *CsaV3_7G035390* in low-nitrogen tolerance in cucumber, a tobacco ringspot virus (TRSV)-based VIGS system was employed. The cucumber phytoene desaturase gene (*CsPDS*) served as a positive control (TRSV: *CsPDS*), resulting in a photo-bleaching phenotype (**Figure 5D**). Plants infected with the empty TRSV vector (TRSV: 00) were used as the negative control. qRT-PCR analysis confirmed that the expression levels of *CsaV3_7G035390* were significantly lower in VIGS plants compared to the negative control (**Figure 5E**), indicating successful silencing of the target gene. Under low-nitrogen treatment for two weeks, silencing of *CsaV3_7G035390* led to significant phenotypic changes. Compared with the TRSV: 00 group, the TRSV: *CsaV3_7G035390* plants exhibited a remarkable reduction in PH and a notable increase in SPAD and nitrogen content of leaf (**Figure 5F**). These results suggest that *CsaV3_7G035390* plays a critical role in nitrogen allocation. The reduction in PH implies that silencing this gene may impair stem growth under nitrogen-deficient conditions. Conversely, the increased SPAD and nitrogen content in the leaves reflect a compensatory mechanism, where nitrogen resources are redistributed to enhance chlorophyll synthesis and sustain photosynthetic activity.

**Table 3:** Annotation and haplotype analysis of 20 candidate genes.

Gene_ID	Haplotype No.	Haplotype traits	Regulation	Significant
CsaV3_1G000260	3	RN_SDW, RN_SPAD	down	yes in F5
CsaV3_1G029650	3	RN_SDW	up	yes in F5
CsaV3_1G036980	3	RN_SDW	up	yes in F5
CsaV3_2G007460	4	RN_SPAD	up	yes in F5
CsaV3_2G013230	2	RN_SDW	up	yes in F5
CsaV3_3G008180	2	RN_SDW	down	yes in F5
CsaV3_3G008170	2	RN_SPAD	up	yes in F5&F27
CsaV3_4G000610	2	RN_SPAD	up	yes in F5
CsaV3_4G000620	2	RN_SPAD	up	yes in F5
CsaV3_4G031970	2	RN_SPAD	up	yes in F5
CsaV3_5G000590	2	RN_SPAD	up	yes in F5
CsaV3_5G028580	2	RN_SPAD	up	yes in F5
CsaV3_5G028620	2	RN_SPAD	up	yes in F5
CsaV3_5G000530	2	RN_SPAD	up	yes in F27
CsaV3_6G038670	2	RN_SPAD	up	yes in F5
CsaV3_7G033020	2	RN_SPAD	down	yes in F5
CsaV3_7G035350	2	RN_SPAD	up	yes in F5
CsaV3_7G035430	2	RN_SPAD	up	yes in F5
CsaV3_7G033010	2	RN_SPAD	up	yes in F5&F27
CsaV3_7G035390	2	RN_SPAD	up	yes in F5&F27

## 4 Discussion

### 4.1 Novel genetic loci related to cucumber low-Nitrogen tolerance at the seedling stage were mined by GWAS

NUE refers to a plant’s ability to effectively absorb and utilize nitrogen under specific nitrogen supply conditions. Plant growth traits, such as PH, SPAD and SDW, are key phenotypic indicators used to assess plant nitrogen response (Guo et al., 2022; Fu et al., 2019; Hou et al., 2021). In this study, a significant reduction in PH, SPAD and SDW were observed in 107 cucumber seedlings after two weeks of low- nitrogen treatment, indicating that nitrogen starvation inhibited cucumber seedling growth and restricted chlorophyll synthesis. This result is consistent with findings in rice (Lv et al., 2021) and maize (Wang et al., 2022). Further analysis revealed that the low-nitrogen tolerant line F005 exhibited a significant growth advantage under LN, with higher PH and SPAD compared to the low-nitrogen sensitive line F027. Additionally, F027 showed noticeable chlorosis after two weeks of LN treatment, in contrast to F005. These results are in agreement with our previous study (Li et al., 2023a), which showed that F005 accumulated significantly more nitrogen in its shoot under LN than F027.

In cucumber GWAS studies, traditional analysis models are commonly used. However, LN tolerance is a complex agronomic trait potentially controlled by multiple loci. Most GWAS methods rely on single-marker analysis, which require stringent *P*-value correction. As a result of these rigorous significance tests, some important association loci may be excluded. In this study, we adopted a 3VmrMLM method based on multi-locus model to avoid false positives. Previous studies have shown that using mrMLM improves the efficiency and robustness of association analysis (Wang et al., 2016). The multi-locus model enhances the power of association analysis, enabling the identification of more loci associated with target traits. a novel GWAS model based on the mrMLM framework, known as 3VmrMLM, has been developed to further interpret genotype effects. Using this approach, we identified 29 QTNs significantly associated with RN_PH, RN_SPAD and RN_SDW, explaining between 2.88% and 15.22% of the phenotypic variation. Specifically, the SNP chr7_22371357, which had an additive effect, and SNP chr6_465156, which had a dominant effect, contributed the most to the RN_SPAD phenotype, accounting for 15.22% and 14.46%, respectively. These loci may represent the most promising candidates for marker-assisted selection (MAS). We compared our findings with previous studies on LN tolerance in cucumber. The RN_PH locus at chr4_22511107 identified in our study is close to the previously reported NAR_9927 and NUpER_9927 (Li et al., 2023a).

### 4.2 Roles of DEGs in low-nitrogen tolerance in cucumber

In this study, we performed transcriptome analysis of cucumber seedlings from low-nitrogen tolerant line F005 and low-nitrogen sensitive line F027 under 10 days of nitrogen deprivation conditions. RNA-seq analysis identified 91 DEGs common to all three comparison groups. GO enrichment analysis revealed the top 20 enriched GO terms for these 91 genes, offering valuable insights into the molecular mechanisms underlying cucumber’s adaptation to LN. Notably, the “cinnamic acid metabolic process in phenylpropanoid metabolism,” a conserved signaling pathway, was significantly enriched in the low nitrogen-treated transcriptome, consistent with previous studies (Zhao et al., 2015), thereby confirming the reliability of our data. Furthermore, enriched pathways such as “urea transport” and “cellular response to nitrate” suggest that these DEGs play a crucial role in nitrogen uptake and utilization. Of particular interest, the *CsNRT2.5* was identified for the first time in this transcriptomic study of long-term nitrogen deprivation, exhibiting significant upregulation across all three comparison groups. The nitrate transporter *AtNRT2.5* is a high-affinity plasma membrane nitrate transporter that plays a critical role in severe nitrogen starvation in adult plants. Under nitrogen starvation, the expression of *AtNRT2.5* is upregulated, and after long-term deprivation, it is most abundant among seven NRT2 family members in the shoots and roots of adult plants (Lezhneva et al., 2014). A growth analysis of multiple NRT2.1, NRT2.2, NRT2.4, and NRT2.5 mutants revealed that *AtNRT2.5*, in conjunction with NRT2.1, NRT2.2, and NRT2.4, ensures efficient nitrate uptake and participates in the phloem loading of nitrate during the redistribution process, supporting the growth of nitrogen-starved adult plants. Ruan et al. (2019), through root morphology, amino acid, and nitrogen-related gene expression analysis, evaluated the response mechanisms of different tea tree varieties to soil nitrogen spatial heterogeneity. They found that the gene *CsNRT2.5*, involved in nitrogen transport and assimilation, was upregulated in nitrogen-efficient varieties and downregulated in inefficient varieties, suggesting that *CsNRT2.5* plays a key role in the adaptation to soil nitrogen spatial heterogeneity in tea trees. Moreover, NRT2.5 has been shown to be expressed in roots, leaves, and seeds in various plants and interacts with NAR2.1 as well as several transcription factors to mediate nitrate signaling (Liu et al., 2020b). These findings suggested that *CsNRT2.5* may play a key role of nitrogen transport in cucumber under LN.

### 4.3 Novel genes related to cucumber low-Nitrogen tolerance at the seedling stage were identified by GWAS and RNA-seq

Thus far, only a few reports have identified the genes associated with cucumber low-Nitrogen tolerance, especially via the GWAS method. Our integrated multi-omics approach uniquely identified 20 high-confidence candidate genes, a strategy not previously reported in cucumber low-nitrogen studies. In the present study, a total of 196 genes were identified in 29 QTN regions for the three low-nitrogen tolerance related traits. To further reduce the number of candidate genes, we integrated the results of the GWAS and RNA-seq analysis, and detected 24 potential genes for the low-nitrogen tolerance traits. Among them, several genes involved in low-nitrogen tolerance that were previously reported in other crops. For example, *TaWRKY46* improves drought resistance in wheat through both ABA-dependent and -independent pathways (Li et al., 2020). *AtWRKY46* regulates lateral root development in Arabidopsis under salt stress (Ding et al., 2014), and *GmWRKY46* negatively regulates phosphorus tolerance in soybean by altering root morphology (Liu et al., 2022). *AtWRKY46* also enhances plant tolerance to ammonium toxicity by regulating protein N-glycosylation and IAA content (Di et al., 2021). In our study, *CsaV3_3G008170* encoding a WRKY46 protein was associated with the RN_SPAD traits, so it was identified as a strong candidate gene. GATA transcription factors, which are evolutionarily conserved, specifically recognize WGATAR sequences. Recent studies have highlighted their role in nitrogen metabolism regulation. For example, Wu et al. (2024) discovered an excellent haplotype, GATA8-H, in modern rice varieties. Under LN, *OsGATA8*-H promotes the expression of *OsAMT3.2*, facilitating ammonium uptake in rice and improving NUE and yield. Under high nitrogen conditions, *OsGATA8*-H also promotes the expression of *OsTCP19*, enhancing the development of effective tillers and reducing ineffective tillers, thereby improving yield and NUE. Zhang et al. (2020) demonstrated that *GmGATA58* is induced by nitrogen levels and plays a key role in regulating chlorophyll synthesis in soybean. Overexpression of *GmGATA58* in the *Arabidopsis thaliana* ortholog *AtGATA21* mutant (gnc) restores the green phenotype by upregulating genes involved in chlorophyll biosynthesis, thereby increasing chlorophyll content and indirectly enhancing the net photosynthetic rate. Therefore, *CsaV3_7G035390*, which encodes the GATA9 protein identified in this study, is also considered a strong candidate gene. In addition, the gene *CsaV3_1G000260* encoding NAC domain-containing protein was identified, and its *Arabidopsis* homolog *AT2G33480* is believed to be related to nitrogen metabolism (Gaudinier et al., 2018), phosphorus metabolism (Hammond et al., 2003), and cold tolerance (Lee et al., 2005).

In addition, two genes involved in ion transport were found to be associated with low-nitrogen tolerance. Wang et al. (2021b) demonstrated that CNGC15 has the function of a calcium ion permeation channel, which interacts with the nitrate receptor NRT1.1 to constitute a molecular switch that, upon the formation or dissociation of the NRT1.1-CNGC15 complex, acts as an ion permeation channel, modulation of calcium channel activity of CNGC15 by sensing nutrient status. The studydemonstrated that CNGC15 has the function of a calcium ion permeable channel, which interacts with the nitrate receptor NRT1.1 to form a molecular switch. When the NRT1.1-CNC15 complex is formed or dissociated, the calcium channel activity of CNGC15 is regulated by sensing the nutritional status. Different nutrients in plants are not independently regulated Research has found that there is a synergistic regulatory mechanism between nitrogen, phosphorus, and potassium to achieve the balance of different nutrients in plants. The Potassium channel AKT1 gene is involved in potassium uptake by plant roots. *OsAKT1* was specifically induced by NO_3_^-^ (Teng et al., 2025). Fang et al. (2020) found that the close relationship between K^+^ and NO3^-^ is mediated by *AtNRT1.1*. We further identified two ion transport-related genes: *CsaV3_5G000590* (encoding a cyclic nucleotide-gated channel) and *CsaV3_1G029650* (encoding a potassium channel AKT1). These findings suggest that ion transport mechanisms may critically contribute to low-nitrogen adaptation in cucumber. We also identified *CsaV3_2G013230* and *CsaV3_5G028620*, encoding a auxin-responsive and Auxin efflux carrier protein that are likely to be involved in the auxin regulatory pathway in cucumber nitrogen metabolism.

In this study, we selected *CsGATA9* as a strong candidate gene for further analysis. The haplotype analysis showed that one SNP existed in the promoters. Based on the SNP, the 107 cucumber accessions were clustered into two haplotypes, Hap.1 with 96 accessions and Hap.2 with 8 accessions. Further correlation analysis showed that the RN_SPAD was significantly higher in Hap.2 than Hap.1, implying that *CsGATA9* might play a vital role in low-nitrogen tolerance. We then knocked-down the expression of *CsGATA9* in cucumber seedlings via VIGS technology, and found that silencing *CsGATA9* led to a reduction in PH, while SPAD and leaf nitrogen accumulation significantly increased. This is consistent with the results of our phenotypic correlation analysis which revealed that a significant negative correlation between PH and SPAD under LN stress (correlation coefficient: −0.09). Similar conclusions have been reported in rice. For example, Liu et al. (2021) conducted large-scale field experiments over three consecutive years under two nitrogen conditions (low and medium nitrogen) on NIL*^OsTCP19^* ^-H^ series and its corresponding recipient parent Kos. The field trials consistently showed that under low and medium nitrogen conditions, NIL*^OsTCP19^* ^-H^ plants exhibited more tillers, higher 1000-grain weight, and shorter PH compared to the Kos plants. These results indicate that the gene regulates plant growth by limiting certain growth processes (such as height increase) and reallocating resources to optimize nitrogen metabolism and growth. However, how *CsGATA9* regulates nitrogen allocation to balance plant growth and nitrogen accumulation remains to be further investigated. To our knowledge, this is the first study to integrate GWAS and RNA-seq for dissecting low-nitrogen tolerance in cucumber, identifying *CsGATA9* as a negative regulator.

The integration of genetics and multi-omics approaches, particularly extending natural variation analysis to molecular mechanisms, is critical for unraveling plant growth, adaptation, and developmental processes (Ahmed et al., 2024). Building on this paradigm, our study synergized GWAS and RNA-seq to analyze low-nitrogen tolerance in cucumber. Through GWAS, we identified multiple QTNs significantly associated with nitrogen stress responses, while RNA-seq profiling under contrasting nitrogen regimes revealed dynamic transcriptional reprogramming in roots. Cross-omics intersection narrowed 196 candidate genes within QTN flanking regions to 24 high-confidence targets, with haplotype analysis further pinpointing 20 key candidates. Crucially, VIGS-mediated silencing of *CsGATA9* confirmed its role in balancing growth suppression (reduced plant height) and nitrogen allocation (enhanced SPAD and leaf nitrogen accumulation) under low nitrogen. Our findings underscore the power of coupling population-scale genetic variation with spatiotemporal transcriptomic dynamics to bridge genotype-phenotype gaps in complex stress tolerance traits.

## 5 Conclusion

Using a panel of 107 cucumber accessions, the low-nitrogen tolerance traits of PH, SPAD and SDW were assessed. Phenotypic characterization analysis revealed significant differences among these three traits. GWAS and RNA-Seq was subsequently employed to map genetic loci associated with nitrogen tolerance phenotypes, and identified 29 QTNs and 20 candidate genes. Of them, *CsGATA9* was experimentally confirmed to play a vital role in low-nitrogen tolerance. These results can provide elite loci and gene resources to aid in the genetic improvement of low-nitrogen tolerance in cucumber.

## Data Availability

The original data in this study can be found in National Center for Biotechnology Information under accession number PRJNA1253396 and the National Genomics Data Center under accession number CRA004282.
