# Supplementary material for: GWAS and RNA-seq reveal novel loci and genes of low-nitrogen tolerance in cucumber (Cucumis sativus L.)
Source: Front Plant Sci. 2025 Jun 6;16:1602360. doi: 10.3389/fpls.2025.1602360 (PMC12179223; doi:10.3389/fpls.2025.1602360)

Supplementary materials

**Supplementary Table 1 | Concentrations of elements of the different nutrient solutions.**

**Supplementary Table 2 | Genes linked to Main-effect QTNs with the traits of RN_PH, RN_SPAD and RN_SDW detected in 107 cucumber accessions.**

**Supplementary Table 3 | Quality statistics of mapping with the reference genome for RNA-Seq.**

**Supplementary Table 4 | Functional Annotation of DEGs in F005 under LN vs. NN.**

**Supplementary Table 5 | Functional Annotation of DEGs in F027 under LN vs. NN.**

**Supplementary Table 6 | Functional Annotation of DEGs in F005 vs. F027 under LN.**

**Supplementary Table 7 | Regulation of 91 common genes across the three comparative groups.**

**Supplementary Table 8 | The Top 20 GO enrichment of the GO analysis.**

**Supplementary Figure 1 | Manhattan Plots of Main-effect QTNs Associated with traits of RN_PH, RN_SPAD and RN_SDW.**


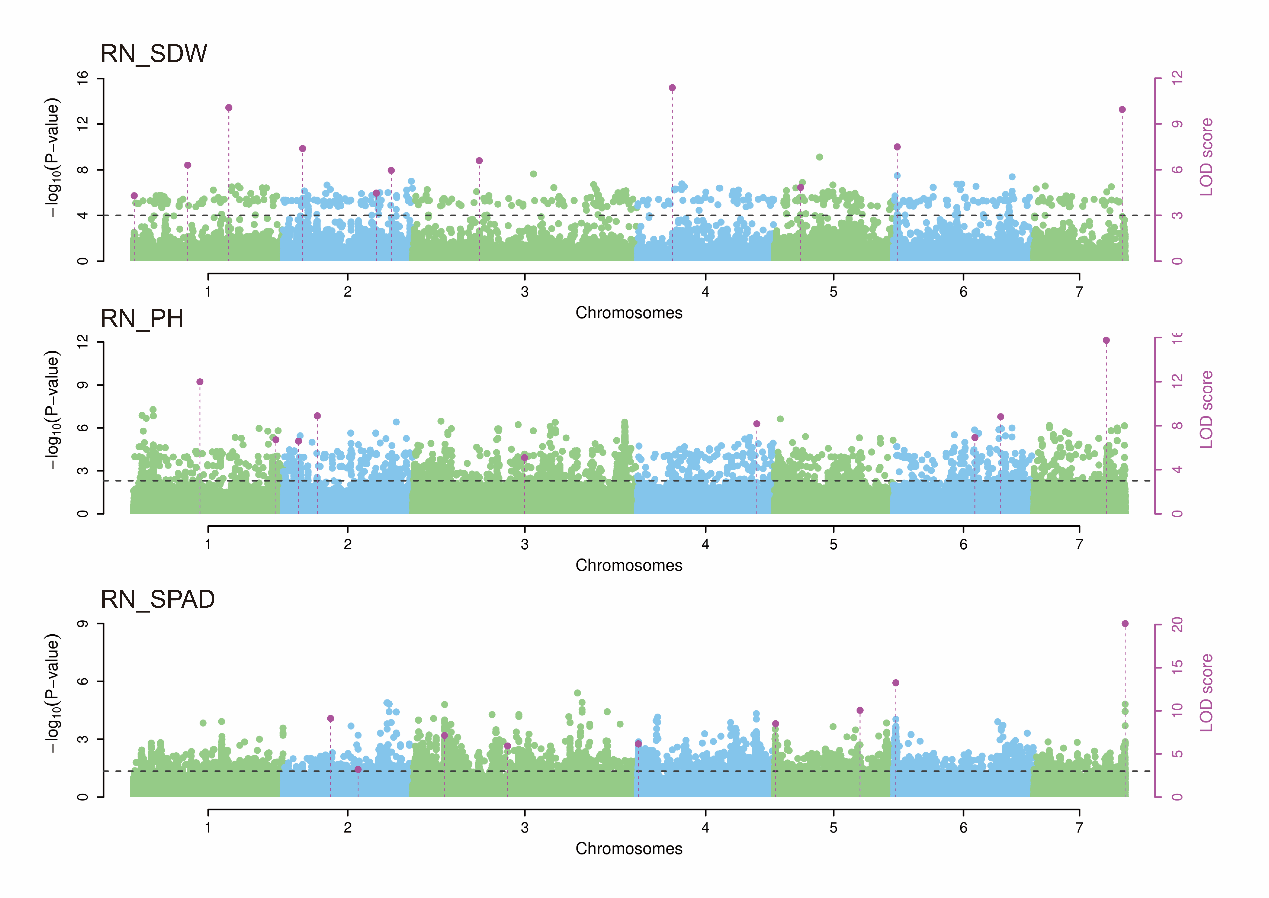

Supplement: Supplementary file 1 [file DataSheet1.docx]
